# Diagnosis and management of Guillain–Barré syndrome in ten steps

**DOI:** 10.1038/s41582-019-0250-9

**Published:** 2019-09-20

**Authors:** Sonja E. Leonhard, Melissa R. Mandarakas, Francisco A. A. Gondim, Kathleen Bateman, Maria L. B. Ferreira, David R. Cornblath, Pieter A. van Doorn, Mario E. Dourado, Richard A. C. Hughes, Badrul Islam, Susumu Kusunoki, Carlos A. Pardo, Ricardo Reisin, James J. Sejvar, Nortina Shahrizaila, Cristiane Soares, Thirugnanam Umapathi, Yuzhong Wang, Eppie M. Yiu, Hugh J. Willison, Bart C. Jacobs

**Affiliations:** 1 Department of Neurology, Erasmus University Medical Center, Rotterdam, Netherlands; 2 Hospital Universitário Walter Cantidio, Universidade Federal do Ceará, Fortaleza, Ceará Brazil; 3 Groote Schuur Hospital, University of Cape Town, Cape Town, South Africa; 4 Department of Neurology, Hospital da Restauração, Recife, Pernambuco Brazil; 5 Department of Neurology, Johns Hopkins University School of Medicine, Baltimore, MD USA; 6 Department of Integrative Medicine, Hospital Universitário Onofre Lopes, Universidade Federal do Rio Grande do Norte, Natal, Brazil; 7 UCL Queen Square Institute of Neurology, University College London, London, UK; 8 International Centre for Diarrhoeal Disease Research, Bangladesh, Dhaka, Bangladesh; 9 Kindai University Faculty of Medicine, Osaka, Japan; 10 Hospital Británico, Buenos Aires, Argentina; 11 Centers for Disease Control and Prevention, Atlanta, GA USA; 12 Department of Neurology, University of Malaya, Kuala Lumpur, Malaysia; 13 Hospital Federal dos Servidores do Estado, Rio de Janeiro, Brazil; 14 National Neuroscience Institute, Singapore, Singapore; 15 Department of Neurology, Affiliated Hospital of Jining Medical University, Jining, Shandong China; 16 Department of Neurology, The Royal Children′s Hospital Melbourne, Melbourne, VIC Australia; 17 Neurosciences Research, Murdoch Children’s Research Institute, Melbourne, VIC Australia; 18 Department of Paediatrics, The University of Melbourne, Melbourne, VIC Australia; 19 College of Medicine, Veterinary and Life Sciences, University of Glasgow, Glasgow, UK; 20 Department of Immunology, Erasmus University Medical Center, Rotterdam, Netherlands

**Keywords:** Autoimmune diseases, Diagnosis, Peripheral neuropathies, Inflammatory diseases

## Abstract

Guillain–Barré syndrome (GBS) is a rare, but potentially fatal, immune-mediated disease of the peripheral nerves and nerve roots that is usually triggered by infections. The incidence of GBS can therefore increase during outbreaks of infectious diseases, as was seen during the Zika virus epidemics in 2013 in French Polynesia and 2015 in Latin America. Diagnosis and management of GBS can be complicated as its clinical presentation and disease course are heterogeneous, and no international clinical guidelines are currently available. To support clinicians, especially in the context of an outbreak, we have developed a globally applicable guideline for the diagnosis and management of GBS. The guideline is based on current literature and expert consensus, and has a ten-step structure to facilitate its use in clinical practice. We first provide an introduction to the diagnostic criteria, clinical variants and differential diagnoses of GBS. The ten steps then cover early recognition and diagnosis of GBS, admission to the intensive care unit, treatment indication and selection, monitoring and treatment of disease progression, prediction of clinical course and outcome, and management of complications and sequelae.

## Abstract (web summary)

In this Consensus Statement, Leonhard et al. provide a globally applicable guideline for the diagnosis and management of Guillain–Barré syndrome, including information on early recognition of the disease, prediction of clinical course and outcome, and management of complications and sequelae.

## Key points

- Classic Guillain–Barré syndrome (GBS) is an acute-onset ascending sensorimotor neuropathy, but the disease can present atypically or as a clinical variant.
- Abnormal results in electrophysiological studies and a combination of an increased protein level and normal cell count in cerebrospinal fluid are classic features of GBS, but patients with GBS can have normal results in both tests, especially early in the disease course.
- Respiratory function should be monitored in all patients as respiratory failure can occur without symptoms of dyspnoea.
- Intravenous immunoglobulin and plasma exchange are equally effective in treating GBS; no other treatments have been proven to be effective.
- The efficacy of repeat treatment in patients who have shown insufficient clinical response is uncertain; nevertheless, this practice is common in patients who show deterioration after an initial treatment response.
- Clinical improvement is usually most extensive in the first year after disease onset and can continue for >5 years.

## Introduction

Guillain–Barré syndrome (GBS) is an inflammatory disease of the PNS and is the most common cause of acute flaccid paralysis, with an annual global incidence of approximately 1–2 per 100,000 person-years^1^. GBS occurs more frequently in males than in females and the incidence increases with age, although all age groups can be affected^1^. Patients with GBS typically present with weakness and sensory signs in the legs that progress to the arms and cranial muscles, although the clinical presentation of the disease is heterogeneous and several distinct clinical variants exist. Diagnosis of GBS is based on the patient history and neurological, electrophysiological and cerebrospinal fluid (CSF) examinations^2–4^. Other diseases that have a similar clinical picture to GBS must be ruled out^4^. Electrophysiological studies provide evidence of PNS dysfunction and can distinguish between the subtypes of GBS: acute inflammatory demyelinating polyradiculoneuropathy (AIDP), acute motor axonal neuropathy (AMAN) and acute motor sensory axonal neuropathy (AMSAN)^5^. Disease progression can be rapid, and most patients with GBS reach their maximum disability within 2 weeks. About 20% of patients with GBS develop respiratory failure and require mechanical ventilation. Cardiac arrhythmias and blood pressure instability can occur owing to involvement of the autonomic nervous system^6^. This involvement of the autonomic nervous system contributes to mortality, which is estimated at 3–10% for patients with GBS even with the best medical care available^7–9^. After the initial progressive phase, patients with GBS reach a plateau phase that can last from days to weeks or months, after which they start to recover, and 60–80% of patients with GBS are able to walk independently 6 months after disease onset, with or without treatment^10,11^. GBS is a monophasic illness, although some patients can deteriorate after first stabilizing or improving on therapy — a phenomenon that is referred to as a treatment-related fluctuation (TRF). Relapses of GBS can occur in 2–5% of patients^10,12–15^.

GBS is thought to be caused by an aberrant immune response to infections that results in damage to peripheral nerves, although the pathogenesis is not fully understood. In a subgroup of patients with GBS, serum antibodies are found against gangliosides, which reside at high densities in the axolemma and other components of the peripheral nerves^16,17^. Complement activation, infiltration of macrophages and oedema are typical characteristics of affected peripheral nerves and nerve roots in patients with GBS^16^.

The incidence of GBS can increase during outbreaks of infectious illnesses that trigger the disease^18^. Most recently, the Zika virus epidemics in French Polynesia in 2013 and in Latin America and the Caribbean in 2015–2016 were linked to an increase in individuals being diagnosed with GBS^19–21^.

The Zika virus outbreaks brought to light the lack of globally applicable guidelines for the diagnosis and management of GBS. Such guidelines are necessary because the diagnosis of GBS can be challenging owing to heterogeneity in clinical presentation, an extensive differential diagnosis, and the lack of highly sensitive and specific diagnostic tools or biomarkers. Guidance for the treatment and care of patients with GBS is also needed because disease progression can vary greatly between patients, which complicates an entirely prescriptive approach to management. In addition, treatment options are limited and costly, and many patients experience residual disability and complaints that can be difficult to manage.

**Fig. 1:**
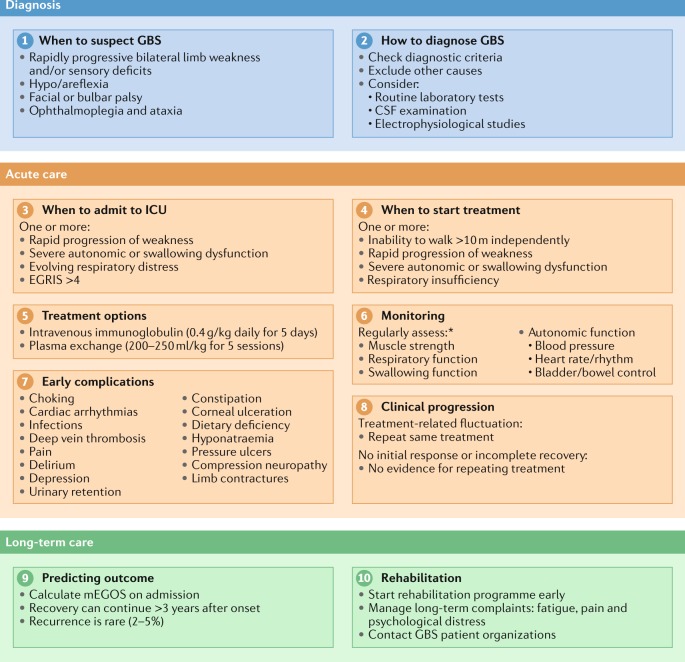
Ten-step approach to the diagnosis and management of Guillain–Barré syndrome. This bullet point summary provides an overview of each of the ten steps described in the guideline. *Frequency of monitoring is dependent on the clinical picture and should be assessed in individual patients. CSF, cerebrospinal fluid; EGRIS, Erasmus GBS Respiratory Insufficiency Score (Box 3); GBS, Guillain-Barré syndrome; ICU, intensive care unit; mEGOS, modified Erasmus GBS Outcome Score (Supplementary Table 3).

Availability of globally applicable clinical guidelines for GBS is especially important as new outbreaks of pathogens that trigger GBS are likely to occur in the future. To generate this globally applicable clinical guideline for GBS, the ten most important steps in the management of GBS, covering diagnosis, treatment, monitoring, prognosis and long-term management, were identified by a group of international experts on GBS (Fig. 1). For each step, recommendations were provided on the basis of evidence from the literature and/or expert opinion, and consensus was sought for each recommendation to finalize the guideline. These recommendations are intended to assist providers in clinical decision-making; however, the use of the information in this article is voluntary. The authors assume no responsibility for any injury or damage to persons or property arising out of or related to any use of this information, or for any errors or omissions.

## Methods

Following the outbreak of Zika virus and its association with an increase in the incidence of GBS, the European Union-funded Zika Preparedness Latin American Network (ZikaPLAN) was established^22^. Our new guideline was initially prepared by participants of the ZikaPLAN network, comprising experts on GBS from the Netherlands (S.E.L., M.R.M. and B.C.J.), Brazil (F.d.A.A.G. and M.E.D.) and the United Kingdom (H.J.W.). These members brought specific clinical and research expertise to the guideline from their leading roles in large international projects on GBS (such as the International GBS Outcome Study (IGOS)), along with direct experience in managing the large increases in GBS cases in Zika virus-affected regions of Latin America^23^. To develop the preliminary guidelines, a series of in-person meetings were held between lead authors on the writing committee (S.E.L., M.R.M., B.C.J. and H.J.W.), along with smaller individual meetings with colleagues in Latin America (S.E.L., F.d.A.A.G. and M.E.D.) and continuous e-mail correspondence to review drafts and receive input. On the basis of their expert opinion and through consensus, this group identified ten of the most important steps in the diagnosis and management of GBS.

For each step, structured literature searches were performed in October 2018 by members of the writing committee (S.E.L and M.R.M), using PubMed and Embase, and the results of these searches provided the basis for the first draft of the guideline. The main inclusion criterion for the literature searches was any study, trial, review or case report published from 2015 onwards that provided detail on the diagnosis, treatment, management or prognosis of patients with GBS. Publications on the pathogenesis of GBS, or those with a focus on diseases not related to GBS, along with publications written in a language other than English or Dutch were excluded from the review. Keywords used in the search strategy included the following Medical Subject Headings (MeSH) terms: “Guillain–Barré syndrome” AND [“diagnosis” OR “therapeutics” OR “treatment outcome” OR “prognosis”]. To obtain literature for more specific topics, additional MeSH terms were combined with primary search keywords, including “intravenous immunoglobulins”, “plasma exchange”, “intensive care units”, “pregnancy”, “Miller Fisher syndrome” and “HIV”. Following this review of the most recent literature, landmark studies published prior to 2015 were identified for inclusion by the writing committee (S.E.L., M.R.M., B.C.J. and H.J.W.), along with additional papers selected by screening the reference lists of already included manuscripts and consultation with the authors. Where possible, our recommendations regarding treatment were based on systematic reviews. Expert opinion from the authors was sought for recommendations when more limited evidence (for example, cohort studies or case–control studies) was available, for instance on topics regarding the differential diagnosis or rehabilitation of GBS.

In consideration of the global variation in healthcare context and variants of GBS, this first draft was subsequently reviewed by an international group of experts on GBS from Argentina (R.R.), Australia (E.M.Y.), Bangladesh (B.I.), Brazil (M.L.B.F. and C.S.), China (Y.W.), Colombia (C.A.P.), Japan (S.K.), Malaysia (N.S.), the Netherlands (P.A.v.D.), Singapore (T.U.), South Africa (K.B.), the United States (D.R.C. and J.J.S.) and the United Kingdom (R.A.C.H). In total, seven rounds of review were held to reach a consensus. To consider the perspective of patients with GBS on the management of the disease, the GBS/CIDP Foundation International, a non-profit organization that provides support, education, research funding and advocacy to patients with GBS or chronic inflammatory demyelinating polyneuropathy (CIDP) and their families, reviewed the manuscript and provided comment during the development of the guideline.

## Step 1: when to suspect GBS

### Typical clinical features

GBS should be considered as a diagnosis in patients who have rapidly progressive bilateral weakness of the legs and/or arms, in the absence of CNS involvement or other obvious causes. Patients with the classic sensorimotor form of GBS present with distal paraesthesias or sensory loss, accompanied or followed by weakness that starts in the legs and progresses to the arms and cranial muscles. Reflexes are decreased or absent in most patients at presentation and in almost all patients at nadir^10,24^. Dysautonomia is common and can include blood pressure or heart rate instability, pupillary dysfunction, and bowel or bladder dysfunction^25^. Pain is frequently reported and can be muscular, radicular or neuropathic^26^. Disease onset is acute or subacute, and patients typically reach maximum disability within 2 weeks^11^. In patients who reach maximum disability within 24 h of disease onset or after 4 weeks, alternative diagnoses should be considered^2,3^. GBS has a monophasic clinical course, although TRFs and relapses occur in a minority of patients^12,13^.

### Atypical clinical presentation

GBS can also present in an atypical manner. Weakness and sensory signs, though always bilateral, can be asymmetrical or predominantly proximal or distal, and can start in the legs, the arms or simultaneously in all limbs^6,26^. Furthermore, severe and diffuse pain or isolated cranial nerve dysfunction can precede the onset of weakness^26^. Young (<6 years old) children in particular can present with nonspecific or atypical clinical features, such as poorly localized pain, refusal to bear weight, irritability, meningism, or an unsteady gait^27,28^. Failure to recognize these signs as an early presentation of GBS might cause delay in diagnosis^28^. In a minority of patients with atypical GBS, particularly those with only motor signs (pure motor variant) and an AMAN subtype on electrophysiological examination, normal or even exaggerated reflexes might be observed throughout the disease course^29^.

### Variants

**Fig. 2:**
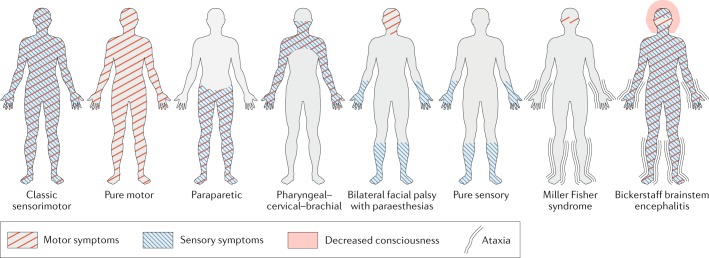
Pattern of symptoms in variants of Guillain–Barré syndrome. Graphic representation of the pattern of symptoms typically observed in the different clinical variants of Guillain–Barré syndrome (GBS). Symptoms can be purely motor, purely sensory (rare) or a combination of motor and sensory. Ataxia can be present in patients with Miller Fisher syndrome and both decreased consciousness and ataxia can be present in patients with Bickerstaff brainstem encephalitis. Symptoms can be localized to specific regions of the body, and the pattern of symptoms differs between variants of GBS. Although bilateral facial palsy with paraesthesias, the pure sensory variant and Miller Fisher syndrome are included in the GBS spectrum, they do not fulfil the diagnostic criteria for GBS. Adapted with permission from ref.^113^, ©2019 BMJ Publishing Group Limited. All rights reserved.

Some patients have a distinct and persistent clinical variant of GBS that does not progress to the classic pattern of sensory loss and weakness. These variants include: weakness without sensory signs (pure motor variant); weakness limited to the cranial nerves (bilateral facial palsy with paraesthesias), upper limbs (pharyngeal–cervical–brachial weakness) or lower limbs (paraparetic variant); and the Miller Fisher syndrome (MFS), which in its full manifestation consists of ophthalmoplegia, areflexia and ataxia^6,30,31^ (Fig. 2 and Table 1). In general, GBS variants are rarely ‘pure’ and often overlap in part with the classic syndrome or show features that are typical of other variant forms^32^.

**Table 1:** Variants of Guillain–Barré syndrome

Variant	Frequency (% of GBS cases)^a^	Clinical features	Refs
Classic sensorimotor GBS^b^	30–85	Rapidly progressive symmetrical weakness and sensory signs with absent or reduced tendon reflexes, usually reaching nadir within 2 weeks	^11, 24, 114, 115^
Pure motor^c^	5–70	Motor weakness without sensory signs	^5, 11, 24^
Paraparetic	5–10	Paresis restricted to the legs	^10, 24, 115^
Pharyngeal–cervical–brachial	<5	Weakness of pharyngeal, cervical and brachial muscles without lower limb weakness	^10, 114, 115^
Bilateral facial palsy with paraesthesias^d^	<5	Bilateral facial weakness, paraesthesias and reduced reflexes	^114– 116^
Pure sensory^d^	<1	Acute or subacute sensory neuropathy without other deficits	^117, 118^
Miller Fisher syndrome	5–25	Ophthalmoplegia, ataxia and areflexia. Incomplete forms with isolated ataxia (acute ataxic neuropathy) or ophthalmoplegia (acute ophthalmoplegia) can occur^31^. Overlaps with classical sensorimotor GBS in an estimated 15% of patients	^11, 24, 114, 116– 119^
Bickerstaff brainstem encephalitis^d^	<5	Ophthalmoplegia, ataxia, areflexia, pyramidal tract signs and impaired consciousness, often overlapping with sensorimotor GBS	^114, 115^

^a^Estimated frequencies, with percentages displayed to the nearest 5%, based on nine (primarily adult) cohort studies in various geographical regions^10,11,24,114–119^. Frequencies differ by region and study, contributing to the variability. Most studies are biased owing to exclusion of some of the variants. ^b^The sensorimotor form is seen in an estimated 70% of patients with GBS in Europe and the Americas, and in 30–40% of cases in Asia^11^. ^c^The pure motor variant is reported in 5–15% of patients with GBS in most studies, but in 70% cases in Bangladesh^11,120^. ^d^Does not fulfil commonly used diagnostic criteria for GBS, which require the presence of bilateral limb weakness or fulfilment of the criteria for Miller Fisher syndrome^3,4^. GBS, Guillain–Barré syndrome.

Besides the variants listed above, pure sensory ataxia, Bickerstaff brainstem encephalitis (BBE) and a pure sensory variant are often included in the GBS spectrum because they share clinical or pathophysiological features with GBS. However, the inclusion of these clinical variants is subject to debate as they do not fulfil the diagnostic criteria for GBS^2,3,31^ (Box 1). The pure sensory variant shares clinical features with the classic sensorimotor form of GBS, with the exception of the presence of motor symptoms and signs^31,33^; pure sensory ataxia and MFS have overlapping clinical profiles, and patients with BBE usually present with symptoms resembling MFS and subsequently develop signs of brainstem dysfunction, including impaired consciousness and pyramidal tract signs^30–32,34–36^. Similar to patients with MFS, individuals with sensory ataxia or BBE can exhibit IgG antibodies to GQ1b or other gangliosides in their serum^30,34^. However, whether pure sensory GBS, pure sensory ataxia and BBE are variants of GBS and/or an incomplete form of MFS is subject to debate, and careful diagnostic work-up is required when these variants are suspected^31,33,35^ (Boxes 1 and 2).

Box 1 Diagnostic criteria for Guillain–Barré syndrome This box lists the diagnostic criteria for Guillain–Barré syndrome (GBS) developed by the National Institute of Neurological Disorders and Stroke (NINDS)^3^ and subsequently modified in a review paper^6^. We have added some features that cast doubt on the diagnosis, which were not mentioned in the original criteria^2,3,6^, and have made some adaptations to improve readability. These criteria are not applicable to some of the specific variants of GBS, as described in Table 1.

**Features required for diagnosis**

- Progressive bilateral weakness of arms and legs (initially only legs may be involved)^a^
- Absent or decreased tendon reflexes in affected limbs (at some point in clinical course)^a^

**Features that strongly support diagnosis**

- Progressive phase lasts from days to 4 weeks (usually <2 weeks)
- Relative symmetry of symptoms and signs
- Relatively mild sensory symptoms and signs (absent in pure motor variant)^a^
- Cranial nerve involvement, especially bilateral facial palsy^a^
- Autonomic dysfunction
- Muscular or radicular back or limb pain^b^
- Increased protein level in cerebrospinal fluid (CSF); normal protein levels do not rule out the diagnosis^b^
- Electrodiagnostic features of motor or sensorimotor neuropathy (normal electrophysiology in the early stages does not rule out the diagnosis)^b^

**Features that cast doubt on diagnosis**

- Increased numbers of mononuclear or polymorphonuclear cells in CSF (>50 × 10^6^/l)
- Marked, persistent asymmetry of weakness
- Bladder or bowel dysfunction at onset or persistent during disease course^b^
- Severe respiratory dysfunction with limited limb weakness at onset^b^
- Sensory signs with limited weakness at onset^a^
- Fever at onset
- Nadir <24 h^b^
- Sharp sensory level indicating spinal cord injury^a^
- Hyper-reflexia or clonus^b^
- Extensor plantar responses^b^
- Abdominal pain^b^
- Slow progression with limited weakness without respiratory involvement
- Continued progression for >4 weeks after start of symptoms^b^
- Alteration of consciousness (except in Bickerstaff brainstem encephalitis)^b^

Minor adaptations were made by the authors to a simplified version of the original NINDS criteria^6^. ^a^Statements in NINDS criteria that were adapted by authors to improve readability. ^b^Additional features which were not included in the NINDS. Note: for clarity, we have omitted ‘Features that rule out the diagnosis’ from the original NINDS criteria for this adapted version.

Box 2 Differential diagnosis of Guillain–Barré syndrome The differential diagnosis of Guillain–Barré syndrome is broad and highly dependent on the clinical features of the individual patient. Here, we present an overview of the most important differential diagnoses categorized by location in the nervous system.

**CNS**

- Inflammation or infection of the brainstem (for example, sarcoidosis, Sjögren syndrome, neuromyelitis optica or myelin oligodendrocyte glycoprotein antibody-associated disorder)^a^
- Inflammation or infection of the spinal cord (for example, sarcoidosis, Sjögren syndrome or acute transverse myelitis)
- Malignancy (for example, leptomeningeal metastases or neurolymphomatosis)
- Compression of brainstem or spinal cord
- Brainstem stroke
- Vitamin deficiency (for example, Wernicke encephalopathy^a^, caused by deficiency of vitamin B1, or subacute combined degeneration of the spinal cord, caused by deficiency of vitamin B12)

**Anterior horn cells**

- Acute flaccid myelitis (for example, as a result of polio, enterovirus D68 or A71, West Nile virus, Japanese encephalitis virus or rabies virus)

**Nerve roots**

- Infection (for example, Lyme disease, cytomegalovirus, HIV, Epstein–Barr virus or varicella zoster virus)
- Compression
- Leptomeningeal malignancy

**Peripheral nerves**

- Chronic inflammatory demyelinating polyradiculoneuropathy (CIDP)
- Metabolic or electrolyte disorders (for example, hypoglycaemia, hypothyroidism, porphyria or copper deficiency)
- Vitamin deficiency (for example, deficiency of vitamins B1 (also known as beriberi), B12 or E)
- Toxins (for example, drugs, alcohol, vitamin B6, lead, thallium, arsenic, organophosphate, ethylene glycol, diethylene glycol, methanol or N-hexane)
- Critical illness polyneuropathy
- Neuralgic amyotrophy
- Vasculitis
- Infection (for example, diphtheria or HIV)

**Neuromuscular junction**

- Myasthenia gravis
- Lambert–Eaton myasthenic syndrome
- Neurotoxins (for example, botulism, tetanus, tick paralysis or snakebite envenomation)
- Organophosphate intoxication

**Muscles**

- Metabolic or electrolyte disorders (for example, hypokalaemia, thyrotoxic hypokalaemic periodic paralysis, hypomagnesaemia or hypophosphataemia)
- Inflammatory myositis
- Acute rhabdomyolysis
- Drug-induced toxic myopathy (for example, induced by colchicine, chloroquine, emetine or statins)
- Mitochondrial disease

**Other**

- Conversion or functional disorder

^a^Differential diagnosis for Bickerstaff brainstem encephalitis.

### Preceding events

About two-thirds of patients who develop GBS report symptoms of an infection in the 6 weeks preceding the onset of the condition^11^. These infections are thought to trigger the immune response that causes GBS^6^. Six pathogens have been temporally associated with GBS in case–control studies: *Campylobacter jejuni*, cytomegalovirus, hepatitis E virus, *Mycoplasma pneumoniae*, Epstein–Barr virus and Zika virus^18,20,37^. It has been suggested that other pathogens are linked to GBS on the basis of evidence from case series or epidemiological studies, but their role in the pathogenesis of GBS is uncertain^38–43^. In general, the absence of an antecedent illness does not exclude a diagnosis of GBS, as putative infections or other immunological stimuli can be subclinical.

Vaccines were first linked to GBS in 1976 when a 7.3-fold increase in the risk of GBS was observed among nonmilitary individuals in the United States who had received the ‘swine’ influenza vaccine^44^. The epidemiological link between other vaccines and GBS has been examined many times since, but only two further studies showed a relationship between GBS and influenza vaccines^45,46^. These studies suggested an increase of approximately one additional GBS case per one million vaccinations, which is several orders of magnitude lower than that observed for the 1976 influenza vaccine^47,48^. No other vaccines have been convincingly linked to GBS^15^.

A relationship between administration of immunobiologicals (for example, tumour necrosis factor antagonists, immune checkpoint inhibitors or type I interferons) and GBS has been reported on the basis of case series information and biological plausibility^49^. Other events, including but not limited to surgery and malignancy, have been temporally related to GBS, but these relationships lack a clear biological rationale and the epidemiological evidence is limited^50,51^.

## Step 2: how to diagnose GBS

In the absence of sufficiently sensitive and specific disease biomarkers, the diagnosis of GBS is based on clinical history and examination, and is supported by ancillary investigations such as CSF examination and electrodiagnostic studies. The two most commonly used sets of diagnostic criteria for GBS were developed by the National Institute of Neurological Disorders and Stroke (NINDS) in 1978 (revised in 1990)^2,3^ (Box 1) and the Brighton Collaboration in 2011 (ref^4^) (Supplementary Table 1). Both sets of criteria were designed to investigate the epidemiological association between GBS and vaccinations but have since been used in other clinical studies and trials. We consider the NINDS criteria to be more suited to the clinician as they present the clinical features of typical and atypical forms of GBS, although the criteria from the Brighton Collaboration are also important, widely used, and can help the clinician to classify cases with (typical) GBS or MFS according to diagnostic certainty. Various differential diagnoses must also be kept in mind when GBS is suspected, and some symptoms should raise suspicion of alternative diagnoses (Boxes 1 and 2). The role of ancillary investigations in confirming a GBS diagnosis is described in more detail in the following section.

### Laboratory investigations

Laboratory testing is guided by the differential diagnosis in individual patients, but in general all patients with suspected GBS will have complete blood counts and blood tests for glucose, electrolytes, kidney function and liver enzymes. Results of these tests can be used to exclude other causes of acute flaccid paralysis, such as infections or metabolic or electrolyte dysfunctions (Box 2). Further specific tests may be carried out with the aim of excluding other diseases that can mimic GBS (Box 2). Testing for preceding infections does not usually contribute to the diagnosis of GBS, but can provide important epidemiological information during outbreaks of infectious diseases, as was seen in previous outbreaks of Zika virus and *C. jejuni* infection^19,52^. The diagnostic value of measuring serum levels of anti-ganglioside antibodies is limited and assay-dependent. A positive test result can be helpful, especially when the diagnosis is in doubt, but a negative test result does not rule out GBS^53^. Anti-GQ1b antibodies are found in up to 90% of patients with MFS^17,54^ and therefore have greater diagnostic value in patients with suspected MFS than in patients with classic GBS or other variants. When GBS is suspected, we advise not to wait for antibody test results before starting treatment.

### Cerebrospinal fluid examination

CSF examination is mainly used to rule out causes of weakness other than GBS and should be performed during the initial evaluation of the patient. The classic finding in GBS is the combination of an elevated CSF protein level and a normal CSF cell count (known as albumino-cytological dissociation)^55^. However, protein levels are normal in 30–50% of patients in the first week after disease onset and 10–30% of patients in the second week^10,11,24,56^. Therefore, normal CSF protein levels do not rule out a diagnosis of GBS. Marked pleocytosis (>50 cells/μl) suggests other pathologies, such as leptomeningeal malignancy or infectious or inflammatory diseases of the spinal cord or nerve roots. Mild pleocytosis (10–50 cells/μl), though compatible with GBS, should still prompt clinicians to consider alternative diagnoses, such as infectious causes of polyradiculitis^10,11^ (Box 2).

### Electrodiagnostic studies

Electrodiagnostic studies are not required to diagnose GBS. However, we recommend that these studies are performed wherever possible as they are helpful in supporting the diagnosis, particularly in patients with an atypical presentation. In general, electrophysiological examination in patients with GBS will reveal a sensorimotor polyradiculoneuropathy or polyneuropathy, indicated by reduced conduction velocities, reduced sensory and motor evoked amplitudes, abnormal temporal dispersion and/or partial motor conduction blocks^6,57^. Typical for GBS is a ‘sural sparing pattern’ in which the sural sensory nerve action potential is normal while the median and ulnar sensory nerve action potentials are abnormal or even absent^6,57^. However, electrophysiological measurements might be normal when performed early in the disease course (within 1 week of symptom onset) or in patients with initially proximal weakness, mild disease, slow progression or clinical variants^5,58,59^. In these patients, a repeat electrodiagnostic study 2–3 weeks later can be helpful. In patients with MFS, results of electrodiagnostic studies are usually normal or demonstrate only a reduced amplitude of sensory nerve action potentials^4,60^.

Electrodiagnostic studies can also differentiate between the three electrophysiological subtypes of classical GBS: AIDP, AMAN, and AMSAN. Several sets of electrodiagnostic criteria exist that aim to classify patients into these different electrophysiological subtypes on the basis of the presence of specific electrodiagnostic characteristics in at least two motor nerves. International consensus is yet to be reached on which set of criteria best defines the electrophysiological subtypes^5,52,61^. However, about one-third of patients with GBS do not meet any of these criteria and are labelled ‘equivocal’ or ‘inexcitable’. Studies have demonstrated that repeating electrodiagnostic studies 3–8 weeks after disease onset might aid electrodiagnostic classification by allowing classification of cases that were initially unclassifiable, or reclassification of cases that were initially classified as AIDP, AMAN or AMSAN, although this practice is controversial^62–64^.

### Imaging

MRI is not part of the routine diagnostic evaluation of GBS, but can be helpful, particularly for excluding differential diagnoses such as brainstem infection, stroke, spinal cord or anterior horn cell inflammation, nerve root compression or leptomeningeal malignancy (Box 2). The presence of nerve root enhancement on gadolinium-enhanced MRI is a nonspecific but sensitive feature of GBS^65^ and can support a GBS diagnosis, especially in young children, in whom both clinical and electrophysiological assessment can be challenging^66^. In light of recent outbreaks of acute flaccid myelitis in young children, the clinical presentation of which can mimic GBS, the potential use of MRI to distinguish between these two diagnoses should be given special attention^67,68^. However, clinicians should be mindful that nerve root enhancement can be found in a minority of individuals with acute flaccid myelitis^69^.

A new potential diagnostic tool in GBS is ultrasound imaging of the peripheral nerves, which has revealed enlarged cervical nerve roots early in the disease course, indicating the importance of spinal root inflammation as an early pathological mechanism^70,71^. This technique might, therefore, help establish a diagnosis of GBS early in the disease course, although further validation is required.

## Step 3: when to admit to the ICU

Reasons to admit patients to the intensive care unit (ICU) include the following: evolving respiratory distress with imminent respiratory insufficiency, severe autonomic cardiovascular dysfunction (for example, arrhythmias or marked variation in blood pressure), severe swallowing dysfunction or diminished cough reflex, and rapid progression of weakness^72,73^. A state of imminent respiratory insufficiency is defined as clinical signs of respiratory distress, including breathlessness at rest or during talking, inability to count to 15 in a single breath, use of accessory respiratory muscles, increased respiratory or heart rate, vital capacity <15–20 ml/kg or <1 l, or abnormal arterial blood gas or pulse oximetry measurements.

As up to 22% of patients with GBS require mechanical ventilation within the first week of admission, patients at risk of respiratory failure must be identified as early as possible^74^. The Erasmus GBS Respiratory Insufficiency Score (EGRIS) prognostic tool was developed for this purpose and calculates the probability (1–90%) that a patient will require ventilation within 1 week of assessment^74^ (Box 3).

Risk factors for prolonged mechanical ventilation include the inability to lift the arms from the bed at 1 week after intubation, and an axonal subtype or unexcitable nerves in electrophysiological studies^75^. Early tracheostomy should be considered in patients who have these risk factors.

Box 3 Erasmus GBS Respiratory Insufficiency Score The Erasmus Guillain–Barré syndrome (GBS) Respiratory Insufficiency Score (EGRIS) calculates the probability that a patient with GBS will require mechanical ventilation within 1 week of assessment and is based on three key measures. Each measure is categorized and assigned an individual score; the sum of these scores gives an overall EGRIS for that patient (between 0 and 7). An EGRIS of 0–2 indicates a low risk of mechanical intervention (4%), 3–4 indicates an intermediate risk of mechanical intervention (24%) and ≥5 indicates a high risk of mechanical intervention (65%). This model is based on a Dutch population of patients with GBS (aged >6 years) and has not yet been validated internationally. Therefore, it may not be applicable in other age groups or populations. An online resource that automatically calculates the EGRIS for a patient based on answers to a series of questions has been made available by the International GBS Outcome Study (IGOS) consortium (see Related links). The Medical Research Council (MRC) sum score is the sum of the score on the MRC scale for: muscle weakness of bilateral shoulder abduction; elbow flexion; wrist extension; hip flexion; knee extension; and ankle dorsiflexion. A higher MRC sum score denotes increased disability, up to a maximum score of 60.

**Table:** 

Measure	Categories	Score
Days between onset of weakness and hospital admission	>7 days	0
	4–7 days	1
	≤3 days	2
Facial and/or bulbar weakness at hospital admission	Absent	0
	Present	1
MRC sum score at hospital admission	60–51	0
	50–41	1
	40–31	2
	30–21	3
	≤20	4
EGRIS	NA	0–7

NA, not applicable. Adapted with permission from ref.^74^, Wiley-VCH.

## Step 4: when to start treatment

Immunomodulatory therapy should be started if patients are unable to walk independently for 10 m (refs^76,77^). Evidence on treatment efficacy in patients who can still walk independently is limited, but treatment should be considered, especially if these patients display rapidly progressive weakness or other severe symptoms such as autonomic dysfunction, bulbar failure or respiratory insufficiency^78–80^. Clinical trials have demonstrated a treatment effect for intravenous immunoglobulin (IVIg) when started within 2 weeks of the onset of weakness and for plasma exchange when started within 4 weeks^76,77^. Beyond these time periods, evidence on efficacy is lacking.

## Step 5: treatment options

### Treatment strategies

IVIg (0.4 g/kg body weight daily for 5 days) and plasma exchange (200–250 ml plasma/kg body weight in five sessions) are equally effective treatments for GBS^76,80^. IVIg and plasma exchange carry comparable risks of adverse events, although early studies showed that plasma exchange was more likely than IVIg to be discontinued^76,81^. As IVIg is also easier to administer and generally more widely available than plasma exchange, it is usually the treatment of choice. Besides IVIg and plasma exchange, no other procedures or drugs have been proven effective in the treatment of GBS. Although corticosteroids would be expected to be beneficial in reducing inflammation and, therefore, disease progression in GBS, eight randomized controlled trials on the efficacy of corticosteroids for GBS showed no significant benefit, and treatment with oral corticosteroids was even shown to have a negative effect on outcome^82^. Furthermore, plasma exchange followed by IVIg is no more effective than either treatment alone and insufficient evidence is available for the efficacy of add-on treatment with intravenous methylprednisolone in IVIg-treated patients^82,83^. In clinical settings where resources are limited, small-volume plasma exchange might be an economical and relatively safe alternative to conventional plasma exchange, but this approach cannot be recommended for general use until its efficacy has been established in further trials^84^.

Antimicrobial or antiviral treatment can be considered in patients with GBS who have an ongoing infection; however, preceding infections have usually resolved before the onset of weakness.

### Specific patient groups

#### GBS variants

Patients with pure MFS tend to have a relatively mild disease course, and most recover completely without treatment within 6 months^85^. Therefore, treatment is generally not recommended in this patient group but patients should be monitored closely because a subgroup can develop limb weakness, bulbar or facial palsy, or respiratory failure^32,80^. The severity of the disease course of BBE justifies treatment with IVIg or plasma exchange, although evidence for the efficacy of treatment in this context is limited^34,85^. For the other clinical variants, no evidence regarding treatment is currently available, although many experts will administer IVIg or plasma exchange^86^.

#### Pregnant women

Neither IVIg nor plasma exchange is contraindicated during pregnancy. However, as plasma exchange requires additional considerations and monitoring, IVIg might be preferred^87–89^.

#### Children

There is no indication that it is necessary to deviate from standard adult practice when treating children with GBS^76,78,90^. Evidence on the relative efficacies of plasma exchange and IVIg in children is limited^90^. However, as plasma exchange is only available in centres that are experienced with its use and seems to produce greater discomfort and higher rates of complications than IVIg in children, IVIg is usually the first-line therapy for children with GBS^91^. Although some paediatric centres administer IVIg as 2 g/kg (body weight) over 2 days, rather than the standard adult regimen of 2 g/kg (body weight) over 5 days, one study indicated that TRFs were more frequent with a 2-day regimen (5 of 23 children) than with the 5-day regimen (0 of 23 children)^78^.

## Step 6: monitoring disease progression

Regular assessment is required to monitor disease progression and the occurrence of complications. First, routine measurement of respiratory function is advised, as not all patients with respiratory insufficiency will have clinical signs of dyspnoea. These respiratory measurements can include usage of accessory respiratory muscles, counting during expiration of one full-capacity inspiratory breath (a single breath count of ≤19 predicts a requirement for mechanical ventilation), vital capacity, and maximum inspiratory and expiratory pressure^73,92^. Clinicians should consider using the ‘20/30/40 rule’, whereby the patient is deemed at risk of respiratory failure if the vital capacity is <20 ml/kg, the maximum inspiratory pressure is <30 cmH_2_O or the maximum expiratory pressure is <40 cmH_2_O (ref^93^). Second, muscle strength in the neck, arms and legs should be assessed using the Medical Research Council grading scale or a similar scale, and functional disability should be assessed on the GBS disability scale (Supplementary Table 2), a widely used tool for documenting GBS disease course^94^. Third, patients should be monitored for swallowing and coughing difficulties. Last, autonomic dysfunction should be assessed via electrocardiography and monitoring of heart rate, blood pressure, and bowel and bladder function.

The nature and frequency of monitoring depends on the rate of deterioration, the presence or absence of autonomic dysfunction, the phase of the disease, and the healthcare setting, and should be carefully assessed in each individual patient. Up to two-thirds of the deaths of patients with GBS occur during the recovery phase and are mostly caused by cardiovascular and respiratory dysfunction^6,7,11^. We therefore advise clinicians to stay alert during this phase and monitor the patient for potential arrhythmias, blood pressure shifts, or respiratory distress caused by mucus plugs. This monitoring is especially important in patients who have recently left the ICU and in those with cardiovascular risk factors.

## Step 7: managing early complications

Complications in GBS can cause severe morbidity and death^95^. Some of these complications, including pressure ulcers, hospital-acquired infections (for example, pneumonia or urinary tract infections) and deep vein thrombosis, can occur in any hospitalized bed-bound patient, and standard-practice preventive measures and treatment are recommended. Other complications are more specific to GBS, for example, the inability to swallow safely in patients with bulbar palsy; corneal ulceration in patients with facial palsy; and limb contractures, ossification and pressure palsies in patients with limb weakness (Table 2). Pain, hallucinations, anxiety and depression are also frequent in patients with GBS, and caregivers should specifically ask patients whether they are experiencing these symptoms, especially if patients have limited communication abilities and/or are in the ICU. Recognition and adequate treatment of psychological symptoms and pain at an early stage is important because these symptoms can have a major impact on the wellbeing of patients. Caregivers should also be aware that patients with GBS, even those with complete paralysis, usually have intact consciousness, vision and hearing. It is important, therefore, to be mindful of what is said at the bedside, and to explain the nature of procedures to patients to reduce anxiety. Adequate management of complications is best undertaken by a multidisciplinary team, which might include nurses, physiotherapists, rehabilitation specialists, occupational therapists, speech therapists and dietitians.

**Table 2:** Important complications of Guillain–Barré syndrome

Complication	When to be alert
Choking	Bulbar palsy
Cardiac arrhythmias	All patients
Hospital-acquired infections (e.g., pneumonia, sepsis or urinary tract infection)	Bulbar and facial palsy, immobility, bladder dysfunction, mechanical ventilation
Pain and tactile allodynia	Limited communication
Delirium	Limited communication
Depression	Limited communication
Urinary retention	All patients
Constipation	Immobility
Corneal ulceration	Facial palsy
Dietary deficiency	Bulbar and facial palsy
Hyponatraemia	All patients
Pressure ulcers	Immobility
Compression neuropathy	Immobility
Limb contractures and ossifications	Severe weakness for prolonged period of time

Important complications of Guillain–Barré syndrome (GBS)^72^. Most of these complications can occur in any patient with GBS, at any time, but the second column shows when they are most likely to occur and/or when to be especially alert.

## Step 8: managing clinical progression

### Insufficient response to treatment

About 40% of patients treated with standard doses of plasma exchange or IVIg do not improve in the first 4 weeks following treatment^80,82^. Such disease progression does not imply that the treatment is ineffective, as progression might have been worse without therapy^6^. Clinicians may consider repeating the treatment or changing to an alternative treatment, but at present no evidence exists that this approach will improve the outcome^96,97^. A clinical trial investigating the effect of administering a second IVIg dose is ongoing^98^.

### Treatment-related fluctuations

TRFs are observed in 6–10% of patients with GBS and are defined as disease progression occurring within 2 months following an initial treatment-induced clinical improvement or stabilization^12,13^. TRFs should be distinguished from clinical progression without any initial response to treatment. The general view is that a TRF indicates that the treatment effect has worn off while the inflammatory phase of the disease is still ongoing. Therefore, patients with GBS who display TRFs might benefit from further treatment, and repeating the full course of IVIg or plasma exchange in these patients is a common practice, although evidence to support this approach is lacking^80^.

### CIDP

In ~5% of patients with GBS, repeated clinical relapses suggest a more chronic disease process, and the diagnosis is changed to acute-onset CIDP^12^. Acute-onset CIDP typically presents with three or more TRFs and/or clinical deterioration ≥8 weeks after disease onset^12^.

## Step 9: predicting outcome

Most patients with GBS, even those who were tetraplegic at nadir or required mechanical ventilation for a long period of time, show extensive recovery, especially in the first year after disease onset^11,99^. About 80% of patients with GBS regain the ability to walk independently at 6 months after disease onset^11^. The probability of regaining walking ability can be calculated in individual patients using the modified Erasmus GBS outcome score (mEGOS) prognostic tool^100^ (Supplementary Table 3).

Despite the generally positive prospects for patients with GBS, death occurs in 3–10% of cases, most commonly owing to cardiovascular and respiratory complications, which can occur in both the acute and the recovery phase^7–9^. Risk factors for mortality include advanced age and severe disease at onset^7^. Long-term residual complaints are also common and can include neuropathic pain, weakness and fatigue^101–103^. However, recovery from these complaints may still occur >5 years after disease onset^103^.

Recurrent episodes of GBS are rare, affecting 2–5% of patients, but this percentage is still higher than the lifetime risk of GBS in the general population (0.1%)^14,15^. Many vaccines carry a warning about GBS, although prior GBS is not a strict contraindication for vaccination. Discussion with experts might be useful for patients who were diagnosed with GBS <1 year before a planned vaccination or who previously developed GBS shortly after receiving the same vaccination. In these patients, the benefits of vaccination for specific illnesses (for example, influenza in elderly individuals) must be weighed against the small and possibly only theoretical risk of a recurrent GBS episode^14^.

## Step 10: planning rehabilitation

Patients with GBS can experience a range of long-term residual problems, including incomplete recovery of motor and sensory function, as well as fatigue, pain and psychological distress^103^. Before the patient is discharged, these possible long-term effects of GBS should be considered and managed^104,105^.

### Physical function

Arranging a rehabilitation programme with a rehabilitation specialist, physiotherapist and occupational therapist is a crucial step towards recovery. Programmes should aim to reduce disability in the early stages of recovery and later to restore motor and sensory function and physical condition to predisease levels^106^. Exercise programmes for patients with GBS, which include range-of-motion exercises, stationary cycling, and walking and strength training, have been shown to improve physical fitness, walking ability and independence in activities of daily living^106^. However, the intensity of exercise must be closely monitored as overwork can cause fatigue^106^.

### Fatigue

Fatigue, unrelated to residual motor deficits, is found in 60–80% of patients with GBS and is often one of the most disabling complaints^107,108^. Other causes should be considered before concluding that fatigue in a patient is a residual result of GBS. As with recovery of physical function, a graded, supervised exercise programme has been shown to be useful in reducing fatigue^109^.

### Pain

Severe pain is reported in at least one-third of patients with GBS 1 year after disease onset and can persist for >10 years^14,26^. Chronic pain in GBS is characterized by muscle pain in the lower back and limbs, painful paraesthesias, arthralgia, and radicular pain. Although the pathogenesis of this pain is not fully understood, muscle pain and arthralgia might be attributable to immobility, and neuropathic pain might be caused by regeneration of, or persistent damage to, small nerve fibres^26^. Management strategies include encouraging mobilization and administering drugs for neuropathic or nociceptive pain^104^.

### Psychological distress

Rapid loss of physical function, often in previously healthy individuals, can be severely traumatic and may cause anxiety and/or depression. Early recognition and management of psychological distress is important in patients with GBS, especially as mental status can influence physical recovery and vice versa; referral to a psychologist or psychiatrist might be beneficial for some patients^110^. Providing accurate information to patients on the relatively good chance of recovery and low recurrence risk (2–5%) can help reduce their fear^11,14^. Connecting patients with others who have had GBS can also help guide them through the rehabilitation process. The GBS/CIDP Foundation International — the international patient association for GBS — and other national organizations can help establish these networks.

## Conclusions

GBS can be a complex disorder to diagnose and manage as the clinical presentation is heterogeneous and the prognosis varies widely between patients. Managing GBS can be especially challenging during outbreaks triggered by infectious disease, as was most recently seen during the Zika virus epidemic. In the absence of an international clinical guideline for GBS, we have developed this consensus guideline for the diagnosis and management of GBS. This guideline was developed by a team of clinical neurologists from around the world and is designed for general applicability in all clinical environments, irrespective of specialist capabilities or availability of resources. The step-by-step design was used to focus attention on the most important issues in GBS and to make the guideline easy to use in clinical practice.

As the field of GBS research develops, and ongoing studies aim to improve diagnostics, treatment and prognostic modelling, this guideline will need to be updated regularly. For example, ultrasound imaging of the peripheral nerves is emerging as a potential diagnostic tool and might require further comment in future versions of this guideline. In relation to treatment, the efficacy of complement inhibitors, IgG-cleaving enzymes and a second course of IVIg is being investigated^78,111,112^. Little is known about how to measure and predict long-term outcome in patients with GBS, and validation studies of known prognostic models (for example, mEGOS and EGRIS) and research into new outcome measures are needed. We intend to seek feedback on this guideline and provide updates based on results from ongoing studies and future research.

To further improve the worldwide management of GBS, we aim to use this consensus report as a basis for the development of online information resources, training material and teaching courses. These resources will be directed towards healthcare workers, including clinical neurologists, as well as patients with GBS and their relatives.

## Supplementary information

Supplementary information
