# Supplementary material for: Diagnosis and management of Guillain–Barré syndrome in ten steps
Source: Nat Rev Neurol. 2019 Sep 20;15(11):671–83. doi: 10.1038/s41582-019-0250-9 (PMC6821638; doi:10.1038/s41582-019-0250-9)
Supplement: Supplementary file 1 — Supplementary information [file 41582_2019_250_MOESM1_ESM.pdf]

nature REVIEWS NEUROLOGY

SUPPLEMENTARY INFORMATION

In format as provided by the authors

Diagnosis and management of Guillain–Barré syndrome in ten steps

Sonja E. Leonhard, Melissa R. Mandarakas, Francisco A. A. Gondim, Kathleen Bateman, Maria L. B. Ferreira, David R. Cornblath, Pieter A. van Doorn, Mario E. Dourado, Richard A. C. Hughes, Badrul Islam, Susumu Kusunoki, Carlos A. Pardo, Ricardo Reisin, James J. Sejvar, Nortina Shahrizaila, Cristiane Soares, Thirugnanam Umapathi, Yuzhong Wang, Eppie M. Yiu, Hugh J. Willison and Bart C. Jacobs

https://doi.org/10.1038/s41582-019-0250-9

Supplementary table 1. Brighton Collaboration Diagnostic Criteria for Guillain–Barré syndrome

Level 1	Level 2	Level 3
Bilateral AND flaccid paralysis of the limbs *AND*	Bilateral AND flaccid paralysis of the limbs *AND*	Bilateral AND flaccid paralysis of the limbs *AND*
Decreased or absent tendon reflexes in weak limbs *AND*	Decreased or absent tendon reflexes in weak limbs *AND*	Decreased or absent tendon reflexes in weak limbs *AND*
Monophasic illness pattern and interval between onset AND nadir of weakness between 12 h and 28 days with subsequent clinical plateau *AND*	Monophasic illness pattern and interval between onset and nadir of weakness between 12 h and 28 days with subsequent clinical plateau *AND*	Monophasic illness pattern, interval between onset and nadir of weakness between 12 h and 28 days, with subsequent clinical plateau *AND*
Electrophysiological findings consistent with Guillian–Barré syndrome (GBS) *AND*	CSF total white cell count <50 cells/µl (with or without CSF protein elevation above laboratory normal value) *OR*	Absence of an identified alternative diagnosis for weakness
Cerebrospinal fluid (CSF) protein level above laboratory normal value AND CSF total white cell count <50 cells/µl) *AND*	If CSF not collected or results not available, electrophysiologic studies consistent with GBS *AND*	
Absence of an identified alternative diagnosis for weakness	Absence of an identified alternative diagnosis for weakness	

These diagnostic criteria are not applicable to all variants of GBS (Table 1), e.g. bilateral facial palsy with paresthesias or pure sensory variant, Miller Fisher syndrome (MFS), or Bickerstaff brainstem encephalitis (BBE). Separate diagnostic criteria for the MFS are part of the Brighton Collaboration Criteria, and can be found in the referred publication. Data from REF[1].

Supplementary table 2. GBS Disability Scale[2,3]

0	Healthy
1	Minor symptoms but capable of running or manual work
2	Able to walk 10 m or more without assistance but incapable of running or manual work
3	Able to walk 10 m or more across an open space with help (stick, appliance or support)
4	Bedridden or chairbound
5	Requiring assisted ventilation (for any part of the day or night)
6	Dead

Guillain–Barré syndrome (GBS) Disability Scale grades functional disability from 0–6. This is a widely used tool to measure clinical course in patients with GBS.

Supplementary table 3. Modified Erasmus GBS Outcome Score (mEGOS)

Prognostic factors	*Categories*	*Score*
Age at onset	≤ 40<br>41-60<br>> 60	0<br>1<br>2
Preceding diarrhea	Absent<br>Present	0<br>1
MRC sum score* at day 7 of hospital admission	60-51<br>50-41<br>40-31<br>30-0	0<br>3<br>6<br>9
mEGOS 0-12		
0-6 = Low risk (>90%)		
7-9 = Intermediate risk (70-85%)		
9-12 = High risk (40-70%)		

Prognostic model based on a Dutch cohort predicting the ability to walk independently at 6 months. A high score corresponds to a high risk of being unable to walk independently at 6 months. This has been validated in a Japanese cohort[5]. Further validation is needed in other populations. Visit http://www.gbsstudies.erasmusmc.nl to calculate the mEGOS for your patient with a web based version of this scale.*MRC sum score: sum of on Medical Research Council scale scores for muscle strength of bilateral shoulder abduction, elbow flexion, wrist extension, hip flexion, knee extension and ankle dorsiflexion. Data from REF[4].

1. Sejvar, J. J. *et al.* Guillain-Barré syndrome and Fisher syndrome: case definitions and guidelines for collection, analysis, and presentation of immunization safety data. *Vaccine* **29**, 599-612, (2011).
2. Hughes, R. A. C., Newsom-Davis, J. M., Perkin, G. D. & Pierce, J. M. Controlled trial of prednisolone in acute polyneuropathy. *Lancet* **312**, 750-753, (1978).
3. Guillain-Barré Syndrome Steroid Trial Group. Double-blind trial of intravenous methylprednisolone in Guillain-Barré syndrome. *Lancet* **341**, 586-590, (1993).
4. Walgaard, C. *et al.* Early recognition of poor prognosis in Guillain-Barré syndrome. *Neurology* **76**, 968-975, (2011).

5. Yamagishi, Y. *et al.* Markers for Guillain-Barré syndrome with poor prognosis: a multi-center study. *J Peripher Nerv Syst* **22**, 433-439, (2017).
